# Physical exercise induces mental flow related to catecholamine levels in noncompetitive, but not competitive conditions in men

**DOI:** 10.1038/s41598-023-41518-2

**Published:** 2023-08-30

**Authors:** István Karsai, Zsófia Nagy, Tamás Nagy, Ferenc Kocsor, András Láng, Emese Kátai, Attila Miseta, Gábor Fazekas, János Kállai

**Affiliations:** 1 Sports and Physical Education Center, Medical School, University of Pécs, Pécs, Hungary; 2 Department of Laboratory Medicine, Medical School, University of Pécs, Pécs, Hungary; 3 Sport and Medicine Research Group, Regenerative Science, Szentágothai Research Center, University of Pécs, Pécs, Hungary; 4 Institute of Psychology, University of Pécs, Pécs, Hungary; 5 Department of Vascular Surgery, University of Pécs, Pécs, Hungary; 6 Department of Behavioral Sciences, Medical School, University of Pécs, Pécs, Hungary

**Keywords:** Biochemistry, Neuroscience, Physiology, Psychology, Endocrinology

## Abstract

The study aimed to reveal physical exercise conditions and catecholamine response-dependent differences while an individual experiences a flow state (FS) following noncompetitive and competitive running drills. Urine laboratory catecholamine levels were measured using a standard clinical method during pre- and post-physical exercises. The noncompetitive task involved intermittent running drills, from an absolute beginning up through exhaustion. Initially, the drill is performed individually then later competing alongside other runners. Twenty-two males (mean age: 40.27; SD: 5.4; min–max: 31–49 years) were selected in accordance to the following criterion: healthy status without using medication, routine forms of training (running, cycling or swimming) ideally performed with regularity, at least three times per week, 45 min per session. During the noncompetitive task, a high FS experience was associated with a low level of catecholamines, (noradrenaline and adrenaline) while in parallel, the high FS was associated with a low concentration of homovallinic acid. During competitive conditions, the FS-related catecholamine level changes have not yet been found. In conclusion, the low concentration of the circulating catecholamines supports the transient hypofrontality hypothesis regarding the FS experiences. Furthermore, synchronized noradrenaline and adrenaline neurosecretion play an essential role in the manifestation and the prolongation of FS in noncompetitive exercise conditions.

## Introduction

According to Nakamura et al., the flow state manifests as an unexpectedly euphoric, uplifting sensation in which a distal environmental event fuses with an individual’s body and personal space. The spatial and temporal cues of the currently executed actions withdraw from the foreground regarding conscious control ushering to a place consumed with an automatic sequence of successive actions. They flow without strong attention control, worrying, and unpleasant efforts^1^. Attention does not respond to environmental cues, and the social demands become marginal, however, realization remains goal-oriented and self-confident^2–4^. The general description of FS contains a positive affective state involving high cognitive flexibility to integrate complex visual scenes and motor actions. The initial starting point of the flow-guided action is a confidently running mental schema. In most cases, the FS manifests in conditions associated with high-level mental or physical performances and intensive physiological reactions while the outcome of the flow-guided behavior remains individually positively evaluated^5^. The flow state definition originates from several interviews among artists, athletes, outdoor enthusiasts, scientists, explorers, and drug-dependent populations. Its factors were measured by self-reported questionnaires. The challenge-skills balance, action-awareness merging, clear goals, concentration on the task execution, withdrawn self-consciousness, experiences of intrinsic reward, and independence in the sense and interpretation of the emotional state are the main characteristics of the FS. The FS features a dual nature; one side is considered a cognitive working model, and the other side is a desired affective state, the flow experience per se^1^.

Under fMRI recording, increased levels of FS were associated with increased activity in the inferior frontal gyrus and putamen, and in contrast, decreased activity was detected in the medial frontal cortex and amygdala^6^. Similar results were reported in EEG, transcranial direct current brain stimulation, and near-infrared spectroscopy studies^7–9^. Additionally, FS was associated with higher theta activities in the frontal brain areas^10^. These results support the transient hypofrontality neurocognitive theory (THT), in which FS was introduced by Dietrich^11^. Due to the current evidence in support of THT, the components of FS can be articulated in a detailed network wherein the dorsolateral prefrontal cortex via the working memory information buffer plays an essential role in the regulation of environmental direct triggers and the affording automatisms from the striatal system. These areas are engaged in the construction of self-reflective references and the conflict-based arousal system of the brain. While individuals maintain their drive toward a goal-directed action, this transient top-down inhibitory system supports them in remaining focused on the task independently from the redundant environmental signals without intensive conscious effort or control^12^. The FS plays a vast role in innovative artistic creativity including several areas of successful athletic performance. However, rapid changes in the circulation of neurotransmitters and the limitation in fMRI use during intensive physical activity restrict revealing the exact relationship of neurotransmitter background regarding FS.

Human and animal studies demonstrate how physical exercises induce changes in dopamine (D), noradrenaline (NA) and adrenaline (A) while contributing to the form of acute cognitive and behavioral responsibility. The catecholamines, NA, A, and dopamine D are the main regulators in the exercise-induced physical, emotional, and cognitive response and the motivation of the goal-directed execution. The dopaminergic system originates from the substantia nigra and the ventral tegmental nucleus and, via the nigrostriatal pathway, assumes its role in the acquisition and the recalling of the subsequent movement schemata^13, 14^. On the other hand, in consideration of the dopaminergic mesolimbic-mesocortical pathway connecting with medial prefrontal cortex is engaged with motivation and regulation of the reward-oriented behavior in which the approaching (to do something) and the anticipated reward (to plane and imagine a goal positioned in a spatial and temporal environment context with routes and maps) are concomitant agents^15^. However, it is worthy to note the component of the catecholamines (CATs) in any case showed a reciprocal regulation from one another depending on the location of the receptors of the brain^16^. Notably, the conditions of the ongoing exercises influence the outcome of the performance, and the manifestation of the FS^7^.

The exercises are considered a behavioral intervention which enhances brain health and plasticity while providing neuroprotection and mental and physical fitness. Successful performance demands exact goal description, personal persistence, and excessive training of motor sequences, all of which serve as the main prerequisite to higher-quality sports performance^17^. A minimal condition during sports activity is to keep the movement and cognitive strategy for automatized control of the action and is the self-confidence and anxiety-free realization of an idea^18^. Following the consciously controlled movements, drilling the conscious reappraisal training in hypnosis or mindfulness mediations are very beneficial to apply for transmitting the part of the movements into a global gestalt. During the reevaluation, the focus is fixed on the automatic running of the acts while the environmental triggers are desensitized or depersonalized. This mental prolusion plays an essential role in the manifestation of the FS, both in rhythmic, physical drills and the construction of motor sequences. Results from fMRI studies support the specific role of the prefrontal attention network in the induction of FS among athletes and outdoor enthusiasts^19^. The FS manifests during several types of athletic activities and in both younger and older athletes^20^. Attention allocation, a relatively effortless deep innovation into the task-related current activity, is the major area in which to understand the FS, the optimal arousal, and athletic performance success^21^.

Sports and regular physical training make use of the multiple mechanisms of the brain’s hemodynamic and neurotransmitter system, which is beneficial in the enhancement of the motoric skills, social and cognitive capabilities and support the physical and psychic health^22–24^. An earlier study focused on hobby runners found marked catecholamine changes in serum concentration and urine metabolites during physical challenges, namely, the exposition to a noncompetitive or competitive exercise; the D, NA and A concentration remains enhanced until task completion^25, 26^. The main proposed question is the way in which this catecholamine undergoes change during exercise among individuals with different degrees of FS following the completion of a noncompetitive or competitive physical exercise. While the body and the mental apparatus adapt to the physical challenges, the regulation of the NA, A and D play a pivotal role in the emotional and motivational aspect of the adaptation and influence the integration of affective, cognitive and motoric functions^27^. The definition of FS, optimal arousal, mental and emotional state to reach excellent physical or mental performance^2^ has different stages during intensive sport activity. The optimal state is depending on trained motoric and cognitive skills that influence the way of attention allocation. In non-competitive conditions, the dominant mental states are enjoyment, enhanced motivation, perceived control, altered perceptions, absorption, and confidence. However, in a competitive environment the concentration on a fixed goal, the quick decisions, increasing effort, and heightened awareness may be beneficial to reach the win^18^. In this condition, the race stress behaves as a clutch, that induces a change in the rate of arousal, and modification in the focus of the attention allocation. The present study is focusing on special motoric, emotional and cognitive changes which are subjectively manifested as an intensive FS experience. Considering the Transient Hypofrontality Theory regarding the FS^14^, the exercise-induced FS may be a manifestation of prefrontal cortex hypoactivity. This suggestion is supported by EEG studies, however, the neurotransmission research, as of yet, has not revealed a coherent concept regarding FS-related CATs associations. The associations between neurotransmitters in the central nervous system and excreted catecholamines in the urine are not fully proven, however there are clinical data that suggests urinary neurotransmitter testing could be a tool to estimate the nervous system function, as in the case of ADHD, PTSD and depression^28–30^. In this study, we suppose excreted CATs (NA, A and D) and their metabolites; Vanillylmandelic acid (VMA) and homovanillic acid (HVA) are associated with the degree of FS which may be manifested both in noncompetitive exercise and competitive exercise. On the other hand, a manifestation of FS varies in rate and patterns dependent upon other personality predispositions including levels of anxiety, the degree of self-confidence, emotional intelligence^31^, and absorption capability-related parasympathetic psychophysiological activation^32^. The FS and the excreted CATs associations will be measured in pre-and post-exercise in noncompetitive and competitive running conditions. The introduction of the condition-dependent flow is grounded in Csikszentmihalyi’s^3^ and Rakei’s et al.^31^ suggestion in which levels of elevated stress, such as competition or anxiety, results in elevated attention to the environmental demand^33^ and in parallel, attenuates the emergence regarding FS experiences. We suppose (Hy1): The high score in FS will be higher in noncompetitive than when compared with competitive running conditions. (Hy2): The high score in the FS may be attributed with lower scores in anxiety and higher scores in self-confidence. Furthermore, (Hy3): as a previous study suggested^26^, the circulated amount of the CATs is associated with the demand for physical effort during exercise in noncompetitive and competitive conditions and will be increased to the end of the task completion, independently from the applied different conditions. The last yet not least hypothesis (Hy4): we suppose the amount of the excreted CATs will be lower in non-competitive than when compared with competitive conditions.

## Results

The measured blood parameters did not show significant correlation between the non-competitive and the competitive running test as published before^26^.

The obtained data alludes to a difference with a large effect size between noncompetitive and competitive conditions in reference to the action-awareness merging (AAM) flow scale. In competitive conditions, the score of the AAM flow was lower than in the noncompetitive condition (Table 1).

**Table 1:** Flow state components in noncompetitive and competitive conditions.

Components of flow state	Flow total Noncomp	Flow total Comp	Flow CSB Noncomp	Flow CSB Comp	Flow AAM Noncomp	Flow AAM Comp
n = 18						
Mean (SD)	64.50 (9.0)	63.77 (7.7)	36.11 (6.3)	37.44 (5.1)	28.38 (3.3)	26.33 (3.1)
Confidence interval	− 2.67–4.12		− 3.76–1.09		0.66–3.44	
t	0.448		− 1.16		3.12	
*p*	0.66		0.263		0.006	
Cohen’s d	0.106		− 0.273		0.735	

After task completion of flow state total and factor scores and differences between noncompetitive (Noncomp) and competitive (Comp) task. Abbreviations: Noncomp: intermitted running task without competition; Comp: intermitted running task with the competitive condition; Flow total: summarized score of flow; challenges-skills balance CSB; action-awareness merging (AAM).

The well-being (mean = 11.47; SD = 3.0), depression (mean = 11.28; SD = 2.4), and perceived life stress (mean = 20.1; SD = 5.9) personality traits compared to the mean scores of a sample of the national population indicated these participants could be considered a healthy sample^34^. Furthermore, higher FS were associated with lower perceived life stress. Moreover, the post-running administered self-reported anxiousness in most of the cases was lower in both noncompetitive and competitive conditions among participants with high flow state experiences. Considering the post-running emotional state, the low somatic anxiety and the high self-confidence was associated with a higher flow state in noncompetitive and competitive situations (Table 2).

**Table 2:** Association analysis between the flow state and trait and state scores.

Trait and state variables controlled by age		Well-being scale (WBI-5)	Beck depression inventory (BDI)	Perceived stress scale (PSS)	PRQ2 som. anx. noncomp post	PRQ2 cog. anx. noncomp post	PRQ2 self-confid. noncomp post
**Noncompetitive condition**							
Flow non- comp	Total	0.202	− 0.416	− 0.447*	− 0.438	− 0.513	0.587*
Flow non- comp	CSB	0.085	− 0.385	− 0.419	− 0.43	− 0.580*	0.543*
Flow non- comp	AAM	0.385	− 0.407	− 0.427	− 0.401	− 0.323	0.605*

Trait and state variables controlled by age		PRQ2 som. anx. comp post	PRQ2 cog. anx. comp post	PRQ2 self-confid. comp post			
**Competitive condition**							
Flow comp	Total	− 0.517*	0.293	0.816**			
Flow comp	CSB	− 0.456	0.301	0.773**			
Flow comp	AAM	− 0.543*	0.24	0.771**			

*p* < 0.05*; *p* < .01**.

The associations of trait and state variables with several characteristics of flow state. Abbreviations: Noncomp: intermitted running task without competition; Comp: intermitted running task with the competitive condition; Flow Total: summarized score of flow: challenges-skills balance (CSB); action-awareness merging (AAM). Post-Race Anxiety Questionnaire (PRQ-2): PRQ2 somatic anxiety (PRQ2 som. anx. comp post), PRQ2 cognitive anxiety (PRQ2 cog. anx. comp post), PRQ2 Self-confidence (PRQ2 self-confid. comp post).

Repeated measures ANOVAs were used to test the effect of time (pre- vs. post-task), condition (noncompetitive vs. competitive), and their interaction (time × condition) on individual and summarized catecholamine levels. Results are presented in Table 4 (for Ms and SDs see Table 3; for the visualization of individual data see Supplement/Fig. 1). In the case of NA and NA + A, a significant main effect of time and a significant time × condition interaction effect were found. This implies the level of NA and NA + A increased from pre-task to post-task, however, the increase was significantly higher in the competitive condition. In the case of A and D, a significant main effect of time was detected. Levels of both A and D increased from pre-task to post-task. In consideration of the combined measures, a significant main effect of time and a significant time × condition interaction effect were found regarding the combined level of A and NA. The significant main effect of time implied a general increase in the combined level of A and NA from pre-task to post-task. However, the significant interaction effect showed this increase was significantly greater in the case of the competitive condition, as when compared to the noncompetitive condition.

**Table 3:** Descriptive statistics of the analyzed catecholamines in pre-and post-phase of non-competitive and competitive conditions.

	Before running test		After running test	
	Mean (SD)	Min–max	Mean (SD)	Min–max
**Non-competitive**				
NA (nmol/L)	24.01 (10.7)	14.1–62.2	43.62 (19.4)	15.8–108.4
A (nmol/L)	7.77 (2.8)	2.4–13.6	13.80 (13.0)	2.9–65.3
D (nmol/L)	100.37 (25.7)	60.5–143.2	120.15 (30.1)	75.5–172.4
NA_A total	51.40 (20.3)	18.2–120.1	57.45 (29.8)	18.8–173.8
CATs total in total	132.75 (31.1)	87.3–191.4	177.60 (50.3)	96.2–312.1
VMA (mg/L)	1.40 (0.3)	08–1.9	1.57 (0.4)	0.8–2.2
HVA (mg/L)	1.76 (0.6)	0.9–3.7	1.42 (0.5)	0.8–2.5
n = 21				
**Competitive**				
NA (nmol/L)	23.05 (12.1)	13.1–68.1	55.27 (24.4)	23.2–122.2
A (nmol/L)	8.67 (6.1)	2.7–24.7	16.28 (9.3)	7.5–43.3
D (nmol/L)	108.51 (34.2)	55.2–171.7	145.94 (49.9)	79.6–246.6
NA_A total	31.72 (15.7)	19.9–84.2	71.56 (31.3)	32.5–165.5
CATs total in total	140.24 (44.3)	76.4–256.0	217.51 (73.4)	112.2–411.2
VMA (mg/L)	1.61 (0.5)	1.1–2.9	1.81 (0.5)	1.1–3.5
HVA (mg/L)	2.13 (0.8)	1.1–4.3	1.57 (0.8)	07–3.5
n = 18				

Urinary catecholamines NA: noradrenaline, A: adrenaline, D dopamine in various periods of sample taking and task conditions. The CATs: catecholamines were cumulated in total scores namely, containing CATs total: contains the current summarized amount of NA, A, and D. The NA_A total contains the current summarized amount of NA and A. Urinary collected metabolites of VMA, HVA, and concentration in pre and post conditions in noncompetitive and competitive tasks have been scheduled.

Concerning the combined level of CATs (Table 4), a significant main effect of time was found. This showed the combined level of A, NA and D increased from pre-task to post-task. In regard to VMA and HVA, a significant main effect of time and the main effect of the condition were found without distinctive interaction between them. In the case of VMA, this meant its levels were higher in the competitive condition, as compared to the noncompetitive condition, and its levels showed a significant increase from pre-task to post-task. Partial η^2^ values indicated large effect sizes with regard to all significant results.

**Table 4:** The effect of condition, time, and their interaction (time ×x condition) on catecholamines.

	Condition		Partial η^2^	Time		Partial η^2^	Time × Condition		Partial η^2^
	F	p		F	p		F	p	
NA	1.211	.286	.067	**33.615**	**<** **.001**	**.664**	**7.934**	**.012**	**.318**
A	0.638	.436	.036	**14.944**	**.001**	**.468**	2.924	.757	.006
D	4.198	.056	.198	**21.947**	**<** **.001**	**.564**	2.155	.160	.113
NA_A total	0.756	.397	.043	**30.577**	**<** **.001**	**.643**	**47.909**	**<** **.001**	**.738**
CATs total	3.454	.081	.169	**29.526**	**<** **.001**	**.635**	4.306	.053	.202
VMA	**4.769**	**.043**	**.219**	**7.272**	**.015**	**.300**	0.001	.973	.000
HVA	**5.535**	**.031**	**.246**	**19.149**	**<** **.001**	**.530**	1.698	.210	.091

Results of repeated measures ANOVAs. Significant effects are highlighted in bold.

Levels of HVA were also higher in the competitive condition, as compared to the noncompetitive condition, however, its levels generally showed a significant decrease from pre-task to post-task. Similar data were reported in a previous study by Nagy et al.^26^.

Pearson’s correlations (Table 5). demonstrated in the noncompetitive conditions that levels of A and NA and also the summarized amount of the A and NA (but not D) was negatively correlated with reported flow state (total score). Similarly, low levels of HVA (D metabolite) were associated with elevated flow state scores in noncompetitive conditions. VMA showed no significant correlation with flow state scores. In competitive conditions, no significant association was found between flow state experience and catecholamines.

**Table 5:** Associations of post task catecholamine levels with post task flow state experience in competitive and non-competitive conditions.

	Non-competitive condition r (*p*)	Competitive condition r (*p*)
A	− .534 (.013)	− .074 (.769)
NA	− .504 (.020)	− .278 (.264)
A + NA	− .562 (.008)	− .238 (.341)
D	− .120 (.605)	.149 (.556)
HVA	− .475 (.029)	.012 (.963)
VMA	− .271 (.234)	− .048 (.849)

Results of Pearson’s correlations are indicated.

## Discussion

This study aimed to explore the relationship between the flow state experience and the excreted catecholamines while participants underwent noncompetitive or competitive standard running tasks. The obtained results indicate that FS manifests both in noncompetitive and competitive tasks yet differs to varying degrees. In comparing noncompetitive vs competitive intermitted running tasks, the degree of the FS and challenges-skills balance CSB scales have not shown any measurable difference. However, the AAM flow state experience showed a lower score in competitive conditions. Interestingly, the obtained results support the **Hy1**, in which the competitive situation and the manifestation of FS, namely the action-awareness merge (AAM) cognitive function is partially inhibited. This result is consonant with earlier findings regarding stress situations (competition) in which the flow in most cases is lower than when compared with anxiety-free conditions^3^. The lower flow in competitive exercise is not inevitable.

Among athletes, mainly during elite sports training, successfully applied methods include hypnosis, meditation, mindfulness, and relaxation techniques to improve the task focusing abilities and also to keep calm during periods of stress in an anxiety-provoking situation. This intervention enhances the chance of the FS while parallels the improvement of task performance and enhancement regarding the FS^18, 19, 35, 36^.

In another course of results, the association analysis between the FS and health-related trait variables revealed FS is associated with low-level general life stress. In examining the relationships between the degree of the FS and positive and negative affective states in post-measured noncompetitive and competitive conditions, the obtained results illustrated both in noncompetitive and competitive conditions, the FS and levels of self-confidence are equally high. On the other side, the high FS is accompanied with a lower level of somatic and in a partially cognitive anxiety state. The FS and state anxiety associations support the **Hy2** and previous data^1, 3, 33^. These correlations indicate anxiety plays a negative while self-confidence play a positive role in the manifestation of the FS. Therefore, anxiety is disadvantageous, however, self-confidence bears an advantageous effect regarding flow embodiment.

The change in pre-and-post-measured CATs in the noncompetitive and competitive conditions is identical to previously reported data^26^ and show how in all conditions, the pre-and post-measured degree of the CATs concluding task completion significantly increased. The time and condition interaction indicate both NA and A play a vast role in exercise-induced neurotransmitters response. These are supporting results regarding **Hy3**.

Considering the urinary collected metabolites, it can be stated, changes regarding VMA showed a consonant enhancing pattern, both in noncompetitive and competitive situations. This results in support by others^25^, however, presently, the nature of the uncontrolled sex-dependent reaction and other cardiovascular and glucose metabolite effects of these systematic changes has not been exactly interpreted.

Distinctively, the HVA concentration was attenuated after performing noncompetitive and competitive conditions. The role of the manifestation of HVA is thought to be a peripheral indicator regarding the central dopaminergic activity^37^ which significantly decreases following mental stress^38^, however, in this population, considering the small number of enrolled participants precludes thoroughly investigating mental stress. Seemingly, our data indicates exercise-induced changes in the urine concentration of the CATs, with the exception of HVA, are working in a unidirectional and synchronized means. Clearly, the HVA depicted a reverted line.

The main aim of this study was to reveal the associations between CATs and the intensity of FS intensity. The conducted analyses indicated in noncompetitive conditions, the lower rate of NA and A, and HVA low concentration in urine samples predicts elevated FS systematically associated with challenge-skill balance and action-awareness merging cognitive functions. A similar association in competitive conditions occurs, in which elevated environmental triggered stress has not yet been detected. This data is aligned with our **Hy4** which indicates the elevated stress attenuates the manifestation of the FS and inhibits the synchronized release of CATs.

Notably, an result obtained by ANOVA revealed NA + A is higher in competitive conditions. We can underline the importance of this data. We suppose the higher NA + A together may be an essential role in the inhibition of the manifestation of FS in competitive conditions. This difference has been demonstrated during the control of the **Hy1**.

The interaction of FS and CATs changing follow diurnal rhythms, situation-specific effects, sex, illness- and stress-dependent and their effect are different in the cortical and mesolimbic areas of the brain^39^. The present research focused on mainly urine samples among healthy males, who underwent different exercise conditions in which trait and state biochemical and behavioral and personality traits were assessed. The CATs sample taking, and the personality stated data, were precisely scheduled early in the evening including all participants. Consequently, the collected data defined in this standard environment and timescale intervals describes characteristics in reference to both the humoral and the psychological state. The neurophysiologic signature of FS as a psychobiological phenomenon is presently developing and raises numerous questions regarding neurocognitive examinations^40^. The discussion in the present context focuses on the explication of the correlative data since the data gathering in this domain is of yet, considerably limited. Competition in front of an audience triggers elevated effort stress which influences performance and potentially alters personal experiences during an ongoing task. Earlier studies showed the intensity of the FS is inhibited in conditions in which negative emotions are present, in most cases, the pressure of the excessive achievement distracts the control of the allocation of attention. Consequently, the attention focus turns from inner actions schemata and instead remains fixed on the environmental requirements and events^3^.

As a result, personal achievement including goals are interrupted. The biopsychological examination regarding this attention process evolved into a different yet partially fitting theory in support of the neuropsychological mechanism of the FS. The transient hypofrontality theory (THT)^14^, implies exercise induces a schema-like regulation unit in which the motor pattern, the sensory inputs, the cognitive design and the autonomic regulation by the intensive activity of the working memory are collected into an integrated schema. These mechanisms reflect the “second nature or deep structure” regarding human cognition and behavior, wherein the goal-directed action is running with low self-awareness, of which, is a beneficial condition for the manifestation of the FS. Other research results provide a detailed description of a variation in the biochemical mechanism of this prefrontal hypofunction manifested with a low serum concentration of CATs. It has been suggested that a routine induces an attenuation in the concentration of NA and A at the same time, the prefrontal activation after finishing the schema configuration is decreased^41, 42^. Our results support this theoretical context and are consonant with the basic idea of the THT hypothesis.

Transient hypofrontality could be a decreasing ability to perceive the risk or identify the negative consequences of the ongoing flow-associated behavior. A higher degree in flow state experiences detected during non-competitive conditions results in challenges in which skills balance activity is dominant. This is a preparatory mental schema forming the training phase of the planned action and has been running at different times and conditions. To paraphrase, it has mentally been executed before it has been implemented in a defined environmental context. In the course of the schema construction, the schema becomes an abstract “blueprint” for execution. Consequently, the implementation automatically runs down if the environmental cues and conditions are available. In this case, an inner motivated scheme is adjusted to a well-defined environmental condition in which the agent is confident and focuses on conducting and integrating a trained design and an environmental demand. This process is defined as a FS^1^ wherein the intrinsic reward is the free running of the constructed schema with an adequate adjustment in a real environment.

The relevance of the low CATs level of urine in the manifestation of the FS appears in our results from the noncompetitive condition. We suggest low circulation catecholamines (CATs) are associated with an intrinsic motivation state wherein a self-relevant, well-laid mental schema fits the environmental challenges. In contrast, during competitive conditions in which an individual’s attention focuses on the environmental challenges, the rivals and to win accolades, the noradrenaline and adrenaline-releasing augment and the FS becomes, in part, inhibited. Parallel with this is the synergistic release of CATs, of which, is disrupted. Our data emphasizes how FS may be manifested in a competitive situation as well, however, its embodiment is dependent upon decreasing the amount of the excretion of NA + A. The intervention which decreases the CATs disruption and the level of NA + A neurotransmitters may originate from a psychological source, such as induced by mindfulness and relaxation techniques or other mental training methods deemed advantageous for the generation of the elevated FS experiences.

In consideration of the above-mentioned interactions between several components of CATs and the limited amount of association with an exercise-induced FS, the hypofrontality hypothesis is partially supported. However, the description of the exact nature of interactions is not yet clearly articulated nor fleshed out.

## Materials and methods

### Participants

Required sample size for repeated measures ANOVAs was calculated using G*Power 3.1^43^ Setting the threshold for rejecting the null hypothesis to α = 0.05, a sample size of at least 23 participants was calculated to detect even small effects (partial η2 = 0.1) with an adequate power of 0.85. During the preselection phase of the investigation, from a group of recreational athletes, volunteers were invited to participate in a physical training-related experiment. The participant’s current health status was screened using the hemodynamic labor assessment and was duly performed by a Doctor of Medicine. ECG and blood pressure were monitored, and heart rhythm detector was implemented to explore potential risk markers for cardiovascular stress. Furthermore, a psychical health-related questionnaire packet was administered regarding depression, perceived life stress and general health status. All participants’ health-related scores remained in the normal range of the normative population sample. Hemodynamic risks were not revealed. Twenty-two males (mean age: 40.27; SD: 5.4; min–max: 31–49 years, body heigh: 178.14; SD: 5.27; min–max: 170–189 cm; body mass: 82.6, SD: 10.11; min–max: 68.8–110 kg; BMI: 25.66; SD: 2.6; min–max: 21.5–30.8 kg/m^2^) were recruited for the study, of whom, were selected based on routine forms of exercise (running, cycling or swimming) at least three times per week for a minimum of 45 min per session. Additional criteria included a healthy status (no known acute or chronic disease) and no use of prescription medications. To ensure homogeneity of the group and considering many gender differences, including hormonal system that may affect the outcome, our study was restricted to men. All volunteers were asked to avoid consumption of hormone-containing foods, avoid alcohol and the use of drugs 24 h prior to conducting the experiment. The participants were asked to restrain from exercising during the day prior to their assessment and only water consumption was allowed. All running tests were executed during the evening hours. Questionnaires were also completed within 15 min prior to and immediately following the running tests. Informed consent was obtained from all subjects. The investigation was a part of a larger set of experiments that approved by the Regional Committee for Research Ethics of the Locale State University (ref. No.: 7162/2018). All methods were performed in accordance with the Declaration of Helsinki, the relevant guidelines and regulations.

### Procedure

The blood and urine laboratory health indicators were measured using standardized clinical method pre- and post-noncompetitive and competitive physical exercises. The noncompetitive task involved a single intermittent indoor run, from an initial starting point up through complete exhaustion. The competitive task contained the same exercise, however, the running was conducted in a competitive environment including other runners and it was hosted in front of an audience. Neither the baseline nor pre- and post-conditions exercise and the health state-related anamnesis did not show illness-related deficiencies among the participants. The heart rate was registered in the pre- and post-phase of the conditions by a Polar Team Pro System. Volunteers were fitted with transmitters positioned just beneath the xiphoid. Blood and urinary samples were taken immediately in both pre- and post-conditions while completing noncompetitive and competitive tasks. The procedure was identical to one used in an earlier study^26^. All exercises were performed in the evening approximately at the same time in all cases. It took on average 15 min.

### Blood samples

The sample taking and evaluation follow a standardized method used systematically throughout our investigations. Prior to and following the running tests within a few minutes, venous blood collections were performed and stored in suitable vacutainers for testing cellular blood parameters, plasma glucose, lactate analysis and routine laboratory blood tests. The subjects were in sitting position while the blood sampling was taken. Plasma and serum parameters were measured as published before^26^.

### Urine samples

Prior to and following the running test, middle stream urine was collected and stored in native vials. All samples were checked using a rapid test (Cybow 10) and were transferred to the laboratory where it were aliquoted and frozen at − 80 ° until further needed. Following thaw, catecholamines and metabolites (D, A, NA, VMA and HVA) levels were detected by Shimadzu Prominence High-Performance Liquid Chromatography (HPLC) system with an Antec Decade SDCTM electrochemical detector. Chromsystems® kit (Chromsystems® from ABL&E-JASCO Hungary, Budapest, Catecholamines in urine—HPLC kit (ref. No.: 6000)) and reverse phase column (Chromsystems® from ABL&E-JASCO Hungary, Budapest, ref. No.: 5100) were used in full compliance to the manufacturer’s recommendations.

VMA and HVA were detected by the same system using Chromsystems® kit (Chromsystems® from ABL&E-JASCO Hungary, Budapest, VMA, HVA, 5-OHIAA in urine—HPLC kit (ref. No.: 1000/B)) and reverse-phase column (ref. No.: 1100/B) in full adherence to the manufacturer’s recommendations. All data were interpreted utilizing the LabSolution program.

### Questionnaires

The Flow State Questionnaire^44^ was established by the phenomenological definition of FS^1^ and contains twenty items including a response sheet with a five-point Likert scale, which ranges from strongly disagree (1), to strongly agree (5). Following the noncompetitive and competitive running tasks, participants were asked to annotate their flow experiences using the response scales. Example for items: “I was absorbed with the task”. “I felt control in the use of my hand.” The total FS score involves two basic elements: the challenges-skills balance (CSB) (Cronbach’s α = 0.921) and the absorptions regarding the task which is the action-awareness merging (AAM) (Cronbach’s α = 0.907) indicating high FS capability in the defined situation. The CSB grounded a stable self-coherence and self-knowledge wherein the individuals possess clear ideas regarding their talents and skills. The motivation to cope with challenges are poised and aligned to their skills. The AAM refers to changes in time and space experiences, the feeling of the absorption of the task completion and the self-perception, automatisms and the diminishing loss in self-control. The internal consistency in the present sample: Cronbach’s α CSB = 0.893; AAM α = 0.833; FS total α = 0.890.

Other personality trait and state assessment methods are the WHO-5 *Well-Being Scale*^45, 46^ involving five aspects which measure the degree of general social, psychical, and physical health Cronbach’s ɑ = 0.793)*. Beck Depression Inventory BDI Short form-H*^47, 48^, contains nine characteristics to measure the level of depression (Cronbach’s ɑ = 0.745). Furthermore, the *Perceived Stress Scale*^49, 50^ was used which contains fourteen aspects to assess the degree of perceived life stress in the recent past (Cronbach’ ɑ = 0.859)*. Post-Race Questionnaire* PRQ^51, 52^ contains eleven items to assess the somatic anxiety, the cognitive anxiety and the current state of self-confidence pre- and post- measurement of athletic forms of exercise (Cronbach’s ɑ = 0.736); somatic anxiety, cognitive anxiety, and self-confidence ɑ = 0.912–0.656.

### Running test

The Multi-Stage 20-m Shuttle Run test (Beep test) was used as a standardized exercise in our study^53, 54^. Running induces an exact, well-documented catecholamine secretion which takes part in the regulation of physiological, behavioral and psychological outcomes regarding provoked stress. The concentration of catecholamines in the plasma or their metabolites in urine is a strong indicator of the individual’s stress sensitivity among trained and untrained individuals including animals^55, 56^. Previous research has documented increasing levels of NA and A are involved in the emotional factor regarding social behavior^57^. Furthermore, NA is considered a dominant modulator in social behavior, and exercise also induces stress linked to D functions^58^.

### Data analysis

Means and standard deviations were used to describe variables. Levels of flow experience were compared with paired sampled t-tests across varied conditions. Associations between measured and computed trait and state variables were tested using Pearson’s correlations. Repeated measures ANOVAs were used to test the effect of time (pre- vs- post-task), condition (non-competitive vs. competitive), and their interaction on measured and summed values of catecholamine levels. Partial eta-squared (η^2^) values were computed as measures of effect size. According to Cohen, benchmarks for small, medium, and large effects were set at 0.01, 0.06, 0.14, respectively^59^. Lastly, Pearson’s correlations were used again to test the association between reported flow experience and levels of catecholamines after the non-competitive condition. SPSS version 22.0 was used for all statistical analyses.

### Limitations

This interpretation remains considerably open to potential questions regarding the role of the D, of which, in association with the CATs, is not yet clearly defined. The role of the HVA during the manifestation of the flow experiences impacts the amount of the metabolite of D, however, this process is not visible. The hemodynamic stress response has not been controlled and we plan to do so, in the upcoming phase of this study. In addition, the present experimental data only represent population of men, thus further studies may be necessary for women.

### Supplementary Information

Supplementary Figure 1.

Supplementary Figure 2.

## Data Availability

Data are available at the OSF repository (https://bit.ly/exercise_flow).
