# Supplementary material for: Physical exercise induces mental flow related to catecholamine levels in noncompetitive, but not competitive conditions in men
Source: Sci Rep. 2023 Aug 30;13:14238. doi: 10.1038/s41598-023-41518-2 (PMC10469213; doi:10.1038/s41598-023-41518-2)
Supplement: Supplementary file 1 — Supplementary Figure 1. [file 41598_2023_41518_MOESM1_ESM.pdf]

Supplement/ Figure 1. **Observed scores and estimated marginal means for repeated measures ANOVAS:** effect of competitiveness and time on serum levels.

A) NA

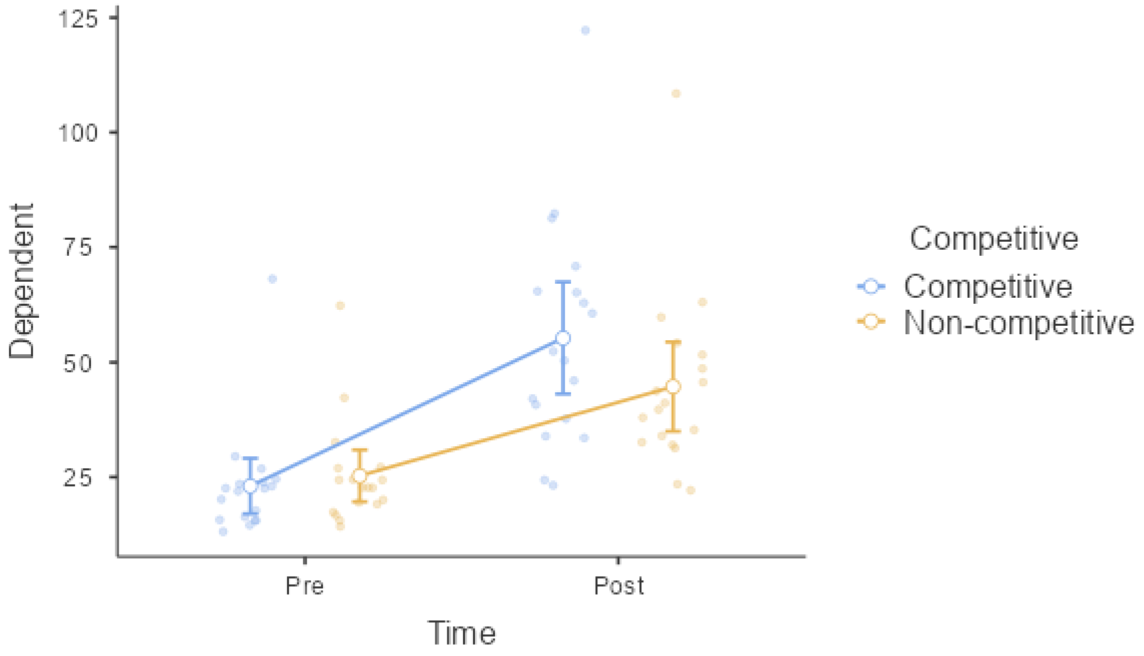

B) A

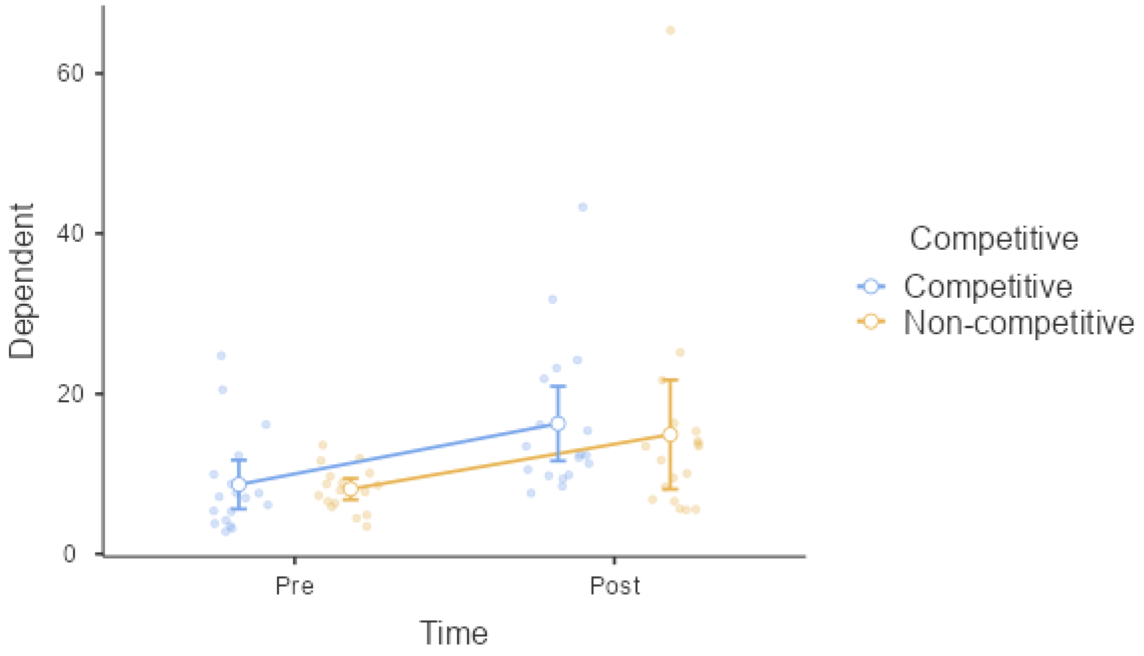

C) D

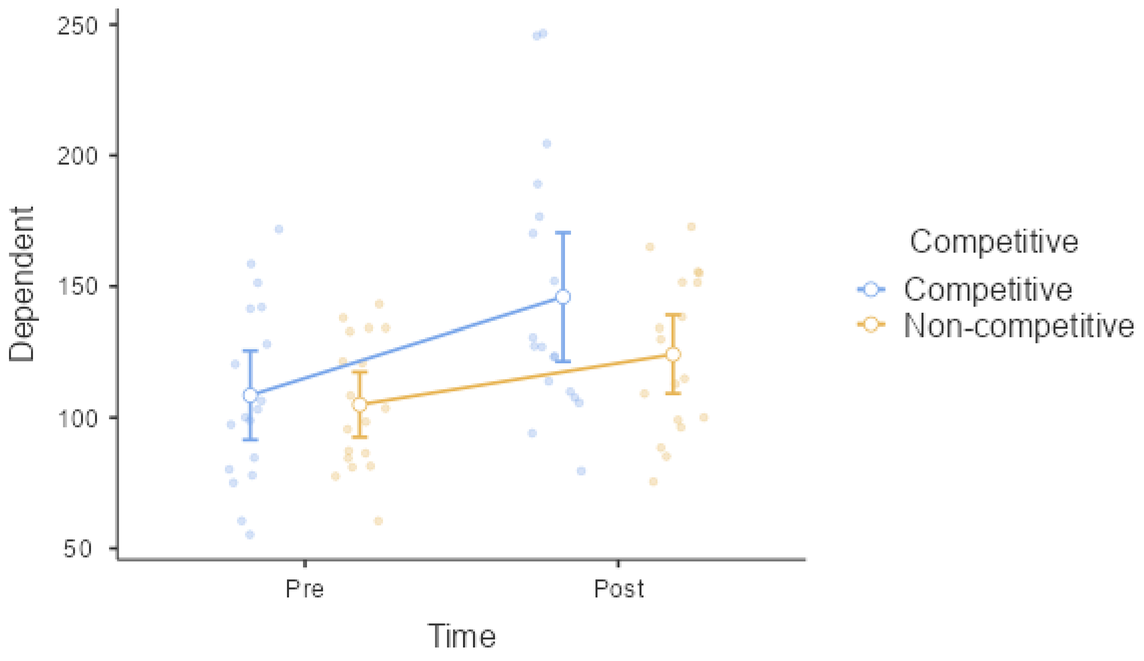
250
200
150
100
50
Dependent
Pre
Post
Time
Competitive
Competitive
Non-competitive

D) NA+A

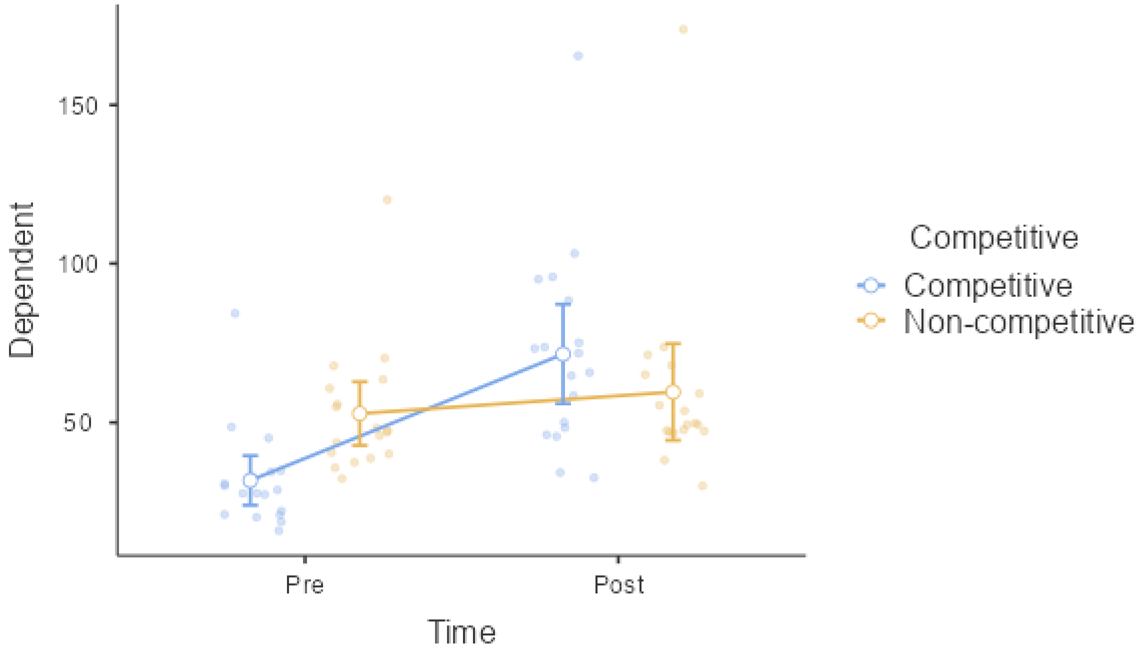
150
100
50
Dependent
Pre
Post
Time
Competitive
Competitive
Non-competitive

E) CATs total

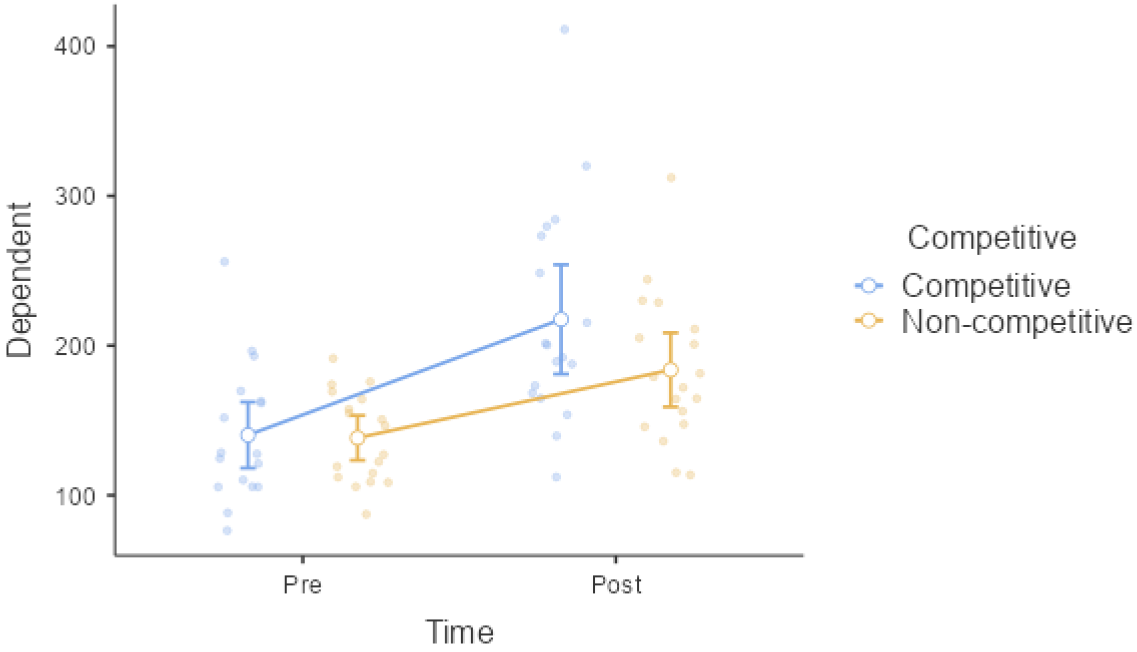

F) VMA

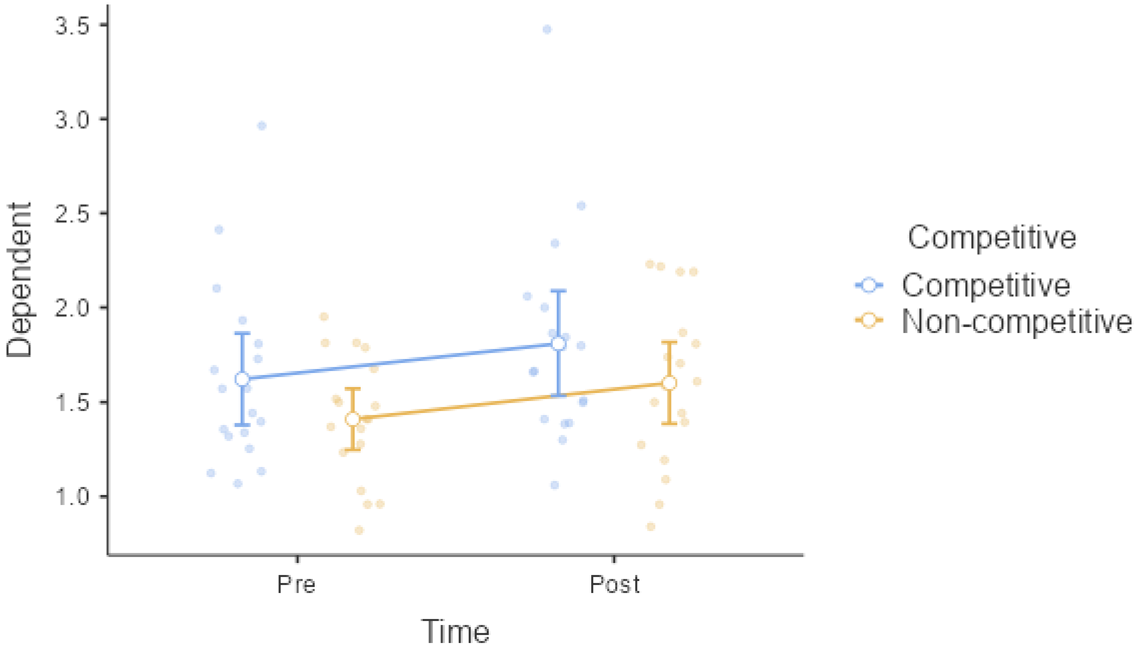

G) HVA

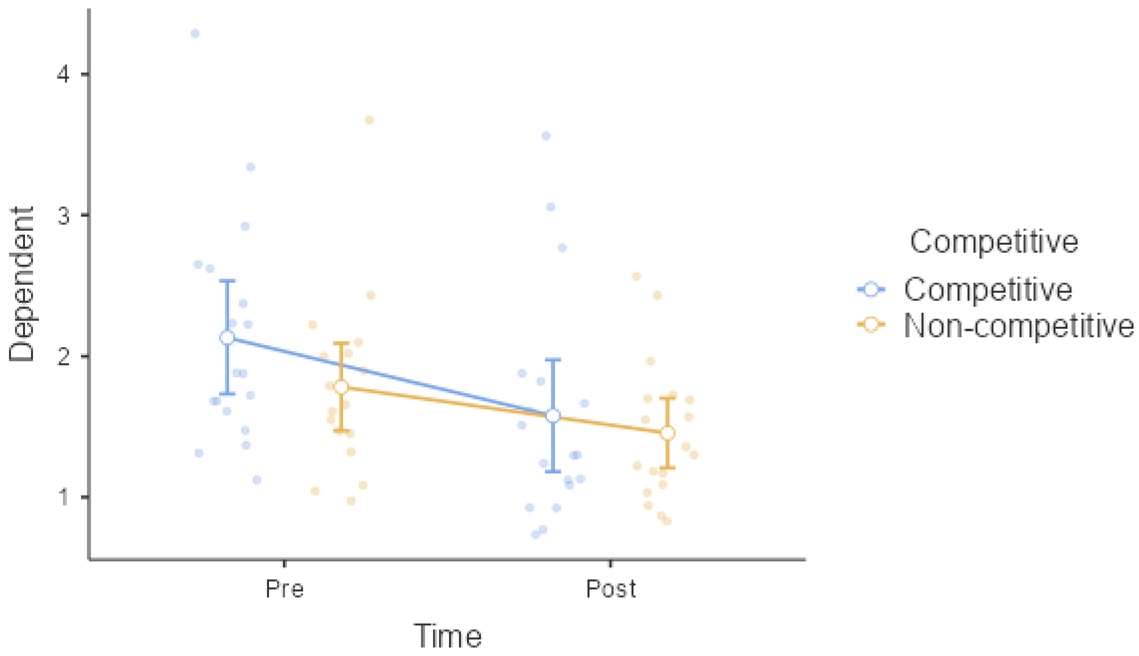

Abbreviations: NA: noradrenaline, A: adrenaline, D: dopamine, CAT: catecholamine, VMA: vanillylmandelic acid, HVA: homovanillic acid.
