# Supplementary material for: Physical exercise induces mental flow related to catecholamine levels in noncompetitive, but not competitive conditions in men
Source: Sci Rep. 2023 Aug 30;13:14238. doi: 10.1038/s41598-023-41518-2 (PMC10469213; doi:10.1038/s41598-023-41518-2)
Supplement: Supplementary file 2 — Supplementary Figure 2. [file 41598_2023_41518_MOESM2_ESM.pdf]

**Physical performance scores following non-competitive and competitive activities**	n	mean (SD)
Non-competitive running test (in meter)	21	2514.8 (569.9)
Competitive running test (in meter)	18	2915.7 (524.4)
Non-competitive running test max pulse (bit/min)	21	182.1 (14.8)
Competitive running test max pulse (bit/min)	18	187.0 (14.5)
Non-competitive plasma lactate (in mmol/L)	21	11.4 (2.5)
Competitive plasma lactate (in mmol/L)	18	10.9 (3.6)

Supplement/ Figure 2.: Participants' health-related variables. Descriptive characteristics of health-realted indexes represent the physical effort of the tasks.

These data were published as :

Nagy, Z. *et al.* Reward Dependence-Moderated Noradrenergic and Hormonal Responses During Noncompetitive and Competitive Physical Activities. *Frontiers in Behavioral Neuroscience* 16, doi:10.3389/fnbeh.2022.763220 (2022).
